# Association between sleep problems and multimorbidity patterns in older adults

**DOI:** 10.1186/s12889-023-15965-5

**Published:** 2023-05-26

**Authors:** Stefany Cristina Claudino Idalino, Jaquelini Betta Canever, Letícia Martins Cândido, Katia Jakovljevic Pudla Wagner, Bruno de Souza Moreira, Ana Lúcia Danielewicz, Núbia Carelli Pereira de Avelar

**Affiliations:** 1 Laboratory of Aging, Resources and Rheumatology, Department of Health Sciences, Federal University of Santa Catarina (UFSC), Campus Araranguá, Rod. Governador Jorge Lacerda, Urussanguinha, Araranguá, 3201, 88906-072 Santa Catarina Brazil; 2 Post-Graduate Program in Neuroscience, Federal University of Santa Catarina (UFSC), Florianópolis, Santa Catarina Brazil; 3 Federal University of Santa Catarina (UFSC), Campus Curitibanos, Rod. Ulysses Gaboardi, 300, Curitibanos, 89520-000 Santa Catarina Brazil; 4 Center for Studies in Public Health and Aging, Federal University of Minas Gerais (UFMG), Av. Alfredo Balena, 190, Santa Efigênia, Belo Horizonte, 30130-100 Minas Gerais Brazil

**Keywords:** Multimorbidity, Older adults, Patterns, Sleep problems

## Abstract

**Background:**

Sleep problems are frequent in older adults and are associated with chronic diseases. However, the association with multimorbidity patterns is still unknown. Considering the negative impacts that multimorbidity patterns can have on older adults’ life, knowledge of this association can help in the screening and early identification of older adults with sleep problems. The objective was to verify the association between sleep problems and multimorbidity patterns in older Brazilian adults.

**Methods:**

This was a cross-sectional study conducted with data from 22,728 community-dwelling older adults from the 2019 National Health Survey. The exposure variable was self-reported sleep problems (yes/no). The study outcomes were: multimorbidity patterns, analyzed by self-report of the coexistence of two or more chronic diseases with similar clinical characteristics: (1) cardiopulmonary; (2) vascular-metabolic; (3) musculoskeletal; (4) coexisting patterns.

**Results:**

Older adults with sleep problems had 1.34 (95%CI: 1.21; 1.48), 1.62 (95%CI: 1.15; 2.28), 1.64 (95%CI: 1.39; 1.93), and 1.88 (95%CI: 1.52; 2.33) greater odds of presenting vascular-metabolic, cardiopulmonary, musculoskeletal, and coexisting patterns, respectively.

**Conclusions:**

These results suggest that public health programs aimed at preventing sleep problems in older adults are essential to reduce possible adverse health outcomes, including multimorbidity patterns and their negative consequences for older adults’ health.

## Background

Sleep problems are frequent in older adults and comprise different disorders, among which insomnia, hypersomnia, circadian rhythm disorders, sleep breathing disorders, narcolepsy, and parasomnias can be highlighted [1]. The prevalence of sleep problems in older adults has been high and this condition affects 32.7–70% of older adults worldwide [2–5] and in up to 50.0% of cases, it may not be diagnosed [6].

Although several studies present sleep problems as an outcome of chronic health conditions [7, 8], sleep problems can lead to multimorbidity in many ways. These include immune system dysregulation [9], since sleep plays an important role in the release of cytokines and proteins, as well as T-cell production, helping to manage inflammation. In addition, sleep problems lead to an imbalance in blood sugar levels [10], insulin sensitivity [11], increased blood pressure, and dysregulation in heart rate [12], which can contribute to the development of heart and metabolic diseases. Sleep problems can also negatively affect mood and mental and brain health [13], increasing the risk of neuropsychiatric conditions such as the development of depression [14], anxiety [15], and dementia [16]. Finally, sleep problems, such as insomnia, can increase daytime fatigue [17] and lead to excessive daytime sleepiness, which increases the risk of falls and other negative mobility-related conditions, reducing individuals’ functioning and predisposing them to the presence of multimorbidity [18].

Multimorbidity is a highly frequent condition in older adults, affecting up to 90% of individuals aged over 60 years [19]. Multimorbidity is a growing public health challenge because it can affect the quality of life of older adults and increase the health care costs [19]. Recent studies have focused on analyzing the different patterns of multimorbidity [20, 21], which are characterized by diseases with similar pathophysiological mechanisms and/or shared risk factors that tend to accumulate in the same individual [19]. Foley et al. [22] suggested that sleep problems are often secondary to comorbidities and not related to aging itself. Recent cross-sectional and longitudinal studies found that sleep problems are associated with several isolated chronic diseases [23–28] and multimorbidity [29–33]. Sindi et al. [34] demonstrated that sleep problems are associated with faster chronic disease accumulation. This result points toward the importance of early detection and treatment of sleep disturbances as a possible strategy to reduce chronic multimorbidity among older adults. However, the association between sleep problems and the different patterns of multimorbidity is still unknown.

Considering the negative impacts that multimorbidity patterns can have on older adults’ life, such as high morbimortality levels and reduced quality of life [35], it is essential to assess how sleep problems are associated with this condition. It is believed that the results of the present study may help in the screening and early identification of older adults with sleep problems and consequently, reduce possible adverse health outcomes in this population. In this context, the aim of this study was to verify the association between sleep problems and multimorbidity patterns in community-dwelling older adults.

## Methods

### Study design and population

This was a cross-sectional study conducted with data from 43,554 community-dwelling older adults from the National Health Survey (PNS, acronym, in Portuguese), carried out in 2019 in Brazil, by the Brazilian Institute of Geography and Statistics (IBGE acronym, in Portuguese), in partnership with the Ministry of Health. The 2019 PNS project was approved by the National Research Ethics Committee (CONEP acronym, in Portuguese) of the National Health Council (CNS acronym, in Portuguese), under process nº 3,529,376, issued in August 2019. This study is in accordance with ethical principles contained in the Declaration of Helsinki.

### Sampling and data collection procedures

The sample of the PNS originated from a master sample, made up of a set of units of areas selected in a register, in order to meet the selection of sub-samples for several different surveys planned by the IBGE, such as National Sample Survey of Households (PNAD acronym, in Portuguese) and Family Budget Survey (POF acronym, in Portuguese). These units are conceptualized as primary sampling units (PSU) within the sampling design of surveys that use the master sample, such as PNS. The second stage consisted of the selection of households by simple random sampling from the National Address Register for Statistical Purposes (CNEFE acronym, in Portuguese), in its most recent update (carried out for the implementation of the Continuous PNAD 2019) before the conclusion of this stage of the sampling plan. Then, within each household, a resident aged *≥* 15 years was randomly selected, based on the list of residents obtained at the time of the interview. To calculate the sample size with the desired level of precision for the estimates, some indicators from the 2013 edition of the PNS were considered, such as data on non-communicable chronic diseases (diabetes mellitus, hypertension, and depression), violence, use of health services, ownership of health insurance, smoking, physical activity, and alcohol consumption, among others.

The 2019 PNS data collection took place between August 2019 and March 2020. The survey sample excluded households located in special census tracts or with low population, such as indigenous groups, barracks, military bases, lodgings, camps, boats, penitentiaries, penal colonies, prisons, jails, long-stay institutions for older adults, networks of integrated care for children and adolescents, convents, hospitals, settlement project villages, and quilombola groups.

The interviews were carried out with mobile collection devices, programmed with the survey questionnaire. Initially, the interviewer made contact with the person in charge or with one of the residents of the selected household. The following were identified: (1) the informant (the one who answered the household questionnaire); (2) all the residents of the household; (3) the resident aged ≥ 15 years (the one who would respond to the individual interview), who was selected through a random selection program on the mobile collection devices. The interviews were scheduled at the most convenient dates and times for the informants, with two or more possible visits to each household. More information about the PNS methodology can be found in the study by Stopa et al. [36]. The database provided by the IBGE presents data on 279,382 individuals, and of these, 43,554 were individuals aged ≥ 60 years.

### Exposure variable

The exposure variable was self-reported sleep problems, assessed through the following question: *“In the past two weeks, how often have you had sleep problems, such as difficulty falling asleep, waking up frequently at night, or sleeping more than usual?”*. The response options were: (1) none of the days; (2) less than half of the days; (3) more than half of the days; (4) almost every day. These responses were recategorized into (1) no sleep problems (answer option 1) and (2) with sleep problems (answer options 2, 3, and 4).

### Outcome variables

The outcomes were multimorbidity patterns, assessed by self-report of the coexistence of two or more chronic diseases with similar clinical characteristics: (1) cardiopulmonary pattern [37]: considering chronic lung diseases (emphysema, chronic bronchitis, and chronic obstructive pulmonary disease) and heart diseases (infarction, angina, heart failure, and others); (2) vascular-metabolic pattern [38]: arterial hypertension, diabetes mellitus, hypercholesterolemia, stroke, cancer, and chronic renal failure (CRF); (3) musculoskeletal pattern [39]: arthritis or rheumatism, chronic back problems, and work-related musculoskeletal disorders; (4) coexisting patterns: the presence of two or more of the aforementioned multimorbidity patterns [40].

### Adjustment variables

The analyses were adjusted for potential confounders described in the literature that theoretically would affect the relationship between sleep problems and multimorbidity patterns in older adults, such as sex (female; male) [41]; age group [42] (60–69; 70–79; ≥ 80 years); schooling [43] (no formal schooling; 1–4; 5–8; 9–11; ≥ 12 years); per capita household income in terms of minimum wages (MW) (< 1 MW; ≥ 1 MW and < 2 MW; ≥ 2 MW) [43]; body mass index [44] (underweight; adequate weight; overweight, considering nutritional status indicators according to Lipschitz [45]; health self-assessment (very good/good; regular; poor/very poor) [46]; the presence of depressive symptoms (score ≥ 10 on the *Patient Health Questionnaire-9*) [47]; adequate consumption of fruits (including juice) and vegetables, considering the recommended consumption of at least 25 portions per week, that is, the sum of these portions, which is approximately equivalent to the daily consumption of five portions of these foods (no; yes) [48]; alcohol consumption assessed from the question: *“How often do you usually consume any alcoholic beverage?”* (I never drink; < 1 time/month; ≥ 1 time/month) [49]; and leisure-time physical activity (insufficiently active; sufficiently active). To classify the older adults as physically active (≥ 150 min/week) or insufficiently active (< 150 min/week) during leisure time, we considered self-reported practices in vigorous physical activities (running/cooper, treadmill running, aerobics/spinning/step/jump, soccer, basketball, or tennis) and in moderate/light physical activities (walking, weight training, water aerobics, localized gymnastics/pilates/stretching/yoga, swimming, martial arts/wrestling, biking, volleyball, or dancing) [50].

### Data analysis

The statistical program STATA version 14.0 (*Stata Corp., College Station*, Texas, EUA) was used in the data analysis. All analyses considered the effect of the study design, incorporating the sample weights by using the *svy* command. Descriptive analysis was performed for all variables, with the calculation of prevalence and respective 95% confidence intervals (95%CI). To test the associations between self-reported sleep problems and multimorbidity patterns, logistic regression analyses were performed, estimating the crude and adjusted *odds ratios* (OR) and their respective 95%CI.

## Results

Of the 43,554 community-dwelling older adults aged ≥ 60 years, 22,728 were included in the analysis because they had data available for all variables, including exposure, outcomes, and adjustment variables. The sample consisted mostly of women (55.3%) and those aged between 60 and 69 years (55.4%). Approximately one-third of the older adults reported studying between 1 and 4 years and slightly less than half of the participants (41.3% [95%CI: 40.5; 42.0]) received less than one minimum wage per capita. Regarding body mass index, 43.6% (95%CI: 42.9; 44.3) had adequate weight, followed by overweight [41.5% (95%CI: 40.8; 42.2)]. As for the health self-assessment, 46.9% (95%CI: 46.2; 47.6) considered their health as very good/good. 90.0% of older adults (95%CI: 89.5; 90.4) did not present depressive symptoms, 61.5% (95%CI: 60.4; 62.6) did not have an adequate consumption of fruit and vegetables, and 73.3% (95%CI: 72.7; 74.0) did not consume alcoholic beverages. Finally, it was observed that most of the sample was insufficiently active (80.6% [95%CI: 80.0; 81.2]) (Table 1).

**Table 1:** Descriptive results of sleep problems, sociodemographic characteristics, health conditions, and lifestyle for the total sample and according to the multimorbidity patterns in older Brazilian adults (N = 22,728). National Health Survey, Brazil, 2019

Variables	Total % (95%CI)	Multimorbidity patterns % (95%CI)			
		Cardiopulmonary % (95%CI)	Vascular-metabolic % (95%CI)	Musculoskeletal % (95%CI)	Coexisting patterns % (95%CI)
**Sleep problems**					
No	58.7 (58.0; 59.4)	1.3 (1.1; 1.5)	27.9 (27.1; 28.7)	6.1 (5.7; 6.6)	3.2 (2.9; 3.6)
Yes	41.2 (40.5; 41.9)	3.6 (3.2; 4.0)	43.0 (41.9; 44.1)	15.2 (14.4; 16.0)	10.5 (9.8; 11.2)
**Sex**					
Male	44.6 (43.9; 45.3)	2.0 (1.7; 2.3)	28.0 (27.0; 29.0)	4.5 (4.1; 4.9)	2.7 (2.3; 3.0)
Female	55.3 (54.6; 56.0)	2.4 (2.1; 2.7)	39.0 (38.1; 40.0)	14.2 (13.6; 14.9)	9.1 (8.5; 9.6)
**Age group**					
60–69 years	55.4 (54.7; 56.1)	1.9 (1.7; 2.2)	31.0 (30.1; 31.8)	9.8 (9.3; 10.4)	5.5 (5.1; 6.0)
70–79 years	31.3 (30.7; 32.0)	2.5 (2.1; 2.9)	39.2 (38.0; 40.4)	10.1 (9.4; 10.9)	7.5 (6.9; 8.3)
≥ 80 years	13.1 (12.6; 13.6)	3.0 (2.4; 3.8)	36.0 (34.1; 37.8)	9.4 (8.3; 10.5)	6.4 (5.5; 7.4)
**Schooling**					
No formal schooling	21.9 (21.3; 22.6)	2.1 (1.7; 2.5)	35.2 (33.7; 36.7)	9.1 (8.3; 9.9)	6.1 (5.4; 6.9)
1–4 years	37.4 (36.7; 38.2)	2.5 (2.1; 2.9)	35.6 (34.4; 36.7)	10.6 (9.9; 11.3)	7.0 (6.4; 7.7)
5–8 years	12.8 (12.3; 13.3)	2.3 (1.8; 3.0)	32.8 (30.9; 34.7)	10.2 (9.1; 11.5)	6.6 (5.7; 7.7)
9–11 years	16.9 (16.3; 17.4)	2.3 (1.8; 2.9)	33.3 (31.6; 34.9)	10.8 (9.8; 11.9)	6.1 (5.3; 7.0)
≥ 12 years	10.7 (10.2; 11.2)	1.6 (1.1; 2.2)	32.0 (30.0; 34.1)	8.1 (7.0; 9.3)	4.8 (3.9; 5.8)
**Per capita household income (minimum wage)**					
< 1	41.3 (40.5; 42.0)	2.3 (2.0; 2.6)	34.9 (33.9; 35.9)	10.3 (9.7; 10.9)	6.7 (6.1; 7.3)
≥ 1 and < 2	30.1 (29.4; 30.8)	2.1 (1.7; 2.5)	34.3 (33.1; 35.6)	10.2 (9.5; 11.0)	6.2 (5.7; 6.9)
≥ 2	28.5 (27.8; 29.3)	2.4 (2.0; 2.8)	33.1 (31.8; 34.4)	8.9 (8.1; 9.6)	5.7 (5.1; 6.3)
**Body mass index**					
Underweight	14.7 (14.2; 15.2)	2.6 (2.1; 3.3)	24.3 (22.8; 25.9)	8.0 (7.1; 9.0)	3.9 (3.2; 4.6)
Adequate weight	43.6 (42.9; 44.3)	1.9 (1.6; 2.2)	30.7 (29.7; 31.7)	8.4 (7.9; 9.0)	4.9 (4.4; 5.3)
Overweight	41.5 (40.8; 42.2)	2.5 (2.2; 2.9)	41.2 (40.1; 42.3)	12.1 (11.4; 12.8)	8.5 (7.9; 9.2)
**Health self-assessment**					
Good/very good	46.9 (46.2; 47.6)	0.8 (0.7; 1.0)	24.1 (23.2; 25.0)	4.9 (4.5; 5.3)	2.3 (2.0; 2.6)
Regular	41.8 (41.1; 42.5)	2.8 (2.4; 3.2)	40.3 (39.3; 41.4)	12.6 (11.8; 13.3)	8.1 (7.5; 8.7)
Poor/very poor	11.2 (10.8; 11.6)	6.1 (5.1; 7.2)	53.3 (51.3; 5.3)	20.6 (19.1; 22.3)	16.0 (14.5; 17.5)
**Depressive symptoms**					
No	90.0 (89.5; 90.4)	1.7 (1.5; 1.9)	31.9 (31.2; 32.6)	8.2 (7.8; 8.6)	4.8 (4.4; 5.1)
Yes	10.0 (9.6; 10.4)	7.2 (6.1; 8.5)	54.2 (52.1; 56.4)	24.9 (23.1; 26.8)	19.5 (17.8; 21.3)
**Adequate consumption of fruits and vegetables**					
No	61.5 (60.4; 62.6)	2.5 (2.1; 2.9)	35.5 (34.2; 36.8)	10.4 (9.7; 11.3)	6.9 (6.2; 7.6)
Yes	38.4 (37.3; 39.5)	2.4 (2.0; 3.0)	35.8 (34.1; 37.5)	11.0 (10.0; 12.2)	6.8 (5.9; 7.7)
**Alcohol consumption**					
I never drink	73.3 (72.7; 74.0)	2.4 (2.1; 2.6)	36.1 (35.3; 36.9)	10.7 (10.2; 11.3)	6.9 (6.5; 7.4)
< 1 time/month	8.5 (8.1; 8.9)	2.3 (1.6; 3.2)	30.2 (28.0; 32.6)	9.8 (8.5; 11.3)	6.1 (5.0; 7.4)
≥ 1 time/month	18.1 (17.5; 18.7)	1.7 (1.3; 2.2)	28.3 (26.7; 29.8)	6.2 (5.5; 7.1)	3.6 (3.0; 4.3)
**Leisure-time physical activity**					
Insufficiently active	80.6 (80.0; 81.2)	2.4 (2.2; 2.7)	34.6 (33.8; 35.4)	10.1 (9.7; 10.6)	6.6 (6.2; 7.0)
Sufficiently active	19.3 (18.7; 19.9)	1.6 (1.2; 2.1)	32.8 (31.3; 34.4)	8.6 (7.7; 9.5)	4.9 (4.3; 5.6)
Number of participants (unweighted)	22,728	456	7,341	2,235	1,326

95%CI: 95% confidence interval

Sleep problems were reported by 41.2% (95%CI: 40.5; 41.9) of the study sample (Table 1). As for multimorbidity patterns, 2.2% (95%CI: 2.0; 2.4) of older adults had a cardiopulmonary pattern, 34.2% (95%CI: 33.5; 34.9) had a vascular-metabolic pattern, 9.8% (95%CI: 9.4; 10.3) had a musculoskeletal pattern, and 6.2% (95%CI: 5.9; 6.6) had coexisting patterns.

In the adjusted analyses of logistic regression, it was observed that older adults with sleep problems had 1.34 (95%CI: 1.21; 1.48), 1.62 (95%CI: 1.15; 2.28), 1.64 (95%CI: 1.39; 1.93), and 1.88 (95%CI: 1.52; 2.33) greater odds of presenting vascular-metabolic, cardiopulmonary, musculoskeletal, and coexisting multimorbidity patterns, respectively, when compared to older adults who did not report sleep problems (Table 2).

**Table 2:** Crude and adjusted analyses of logistic regression between sleep problems and multimorbidity patterns in older Brazilian adults (N = 22,728). National Health Survey, Brazil, 2019

Sleep problems	Multimorbidity patterns							
	Cardiopulmonary		Vascular-metabolic		Musculoskeletal		Coexisting patterns	
	Crude	Adjusted#	Crude	Adjusted#	Crude	Adjusted#	Crude	Adjusted#
	**OR**	**OR**	**OR**	**OR**	**OR**	**OR**	**OR**	**OR**
	**(95%CI)**	**(95%CI)**	**(95%CI)**	**(95%CI)**	**(95%CI)**	**(95%CI)**	**(95%CI)**	**(95%CI)**
Yes	**2.79 (2.26; 3.44)**	**1.62 (1.15; 2.28)**	**1.94 (1.83; 2.06)**	**1.34 (1.21; 1.48)**	**2.75 (2.50; 3.03)**	**1.64 (1.39; 1.93)**	**3.52 (3.08; 4.01)**	**1.88 (1.52; 2.33)**

In bold: statistically significant association. # adjusted for sex, age group, schooling, per capita household income, body mass index, health self-assessment, depressive symptoms, adequate consumption of fruits and vegetables, alcohol consumption, and leisure-time physical activity; OR: odds ratio; 95%CI: 95% confidence interval

## Discussion

The results of the present study showed that older adults with sleep problems were more likely to have multimorbidity patterns (cardiopulmonary, vascular-metabolic, and musculoskeletal) and coexisting patterns when compared to older adults who did not report the presence of these problems.

In this study, older adults with sleep problems were 34% more likely to have a vascular-metabolic multimorbidity pattern, corroborating previous findings that demonstrated that sleep disorders are important risk factors for cardiovascular morbidity and mortality [51]. Evidence suggests that psychosocial factors that increase the risk of developing cardiovascular disease, including increased blood pressure and arterial stiffness, are also related to sleep [52]. Furthermore, satisfactory sleep duration not only maintains adequate body function but also prevents adverse cardiovascular outcomes such as cardiometabolic diseases [53]. It is suggested that sleep has important homeostatic functions, such as suppressive effects on the stress system and the inflammatory system, which are described as pathophysiological mechanisms in the activation of the sympathetic nervous system and of pro-inflammatory pathways that lead to activation of the hypothalamic-adrenal and sympathetic nervous system, predisposing to the hypertensive state [54]. Furthermore, Liu et al. [55] observed that older females with difficulty falling asleep have increased levels of LDL (low-density lipoprotein) cholesterol, which is widely known to increase the risk of developing coronary heart disease [56]. Other recent studies have also linked sleep problems to high total cholesterol and triglyceride levels [57]. One of the main mechanisms involved in sleep problems refers to lipid abnormalities induced by intermittent hypoxia [58], commonly seen in obstructive sleep apnea. Review studies point to the existence of an independent association between obstructive sleep apnea and dyslipidemia [59] and pieces of evidence from a murine model showed that intermittent hypoxia causes hyperlipidemia and upregulation of lipid biosynthesis genes in the liver [60].

The association between sleep problems and chronic lung diseases is still poorly studied, and most of the evidence found to date presents sleep problems and/or their adverse effects as consequences of already-established lung diseases [61]. However, sleep problems are also known to affect breathing through disturbances in respiratory control, respiratory muscle function, and lung mechanics [62]. Negative effects on respiratory control include decreased cortical inputs to the respiratory center with reduced production of neurotransmitters by the respiratory motor neuron and decreased sensitivity of chemoreceptors that affect ventilatory responses to hypoxia and hypercapnia, in addition to increased upper airway resistance [62].

In the present study, older adults with sleep problems were 64% more likely to have a musculoskeletal multimorbidity pattern. Among the diseases that compose this pattern, uncontrolled rheumatoid arthritis is the one most closely related to reduced sleep quality [63], suggesting that insufficient sleep may exacerbate pain in patients with rheumatoid arthritis. A recent longitudinal study [64] found that sleep problems can be considered risk factors for the development of rheumatoid arthritis. The authors observed a 49% higher risk of developing rheumatoid arthritis among those with sleep problems compared to the population without the same complaint. Similarly, a cohort study [65] with a one-year follow-up conducted with 1,955 health professionals (60% nurses) found a strong association between sleep problems and the development of low back pain, with an odds ratio of 3.16 (95%CI: 1.93; 5.17).

In this study, older adults who reported sleep problems were 88% more likely to have the coexistence of multimorbidity patterns. Similar results were found by Lima et al. [66], who analyzed data from 85,531 Brazilians (≥ 18 years) and verified the existence of an association between self-reported sleep problems and the presence of non-communicable chronic diseases and multimorbidities. A possible explanation for this association may be related to the fact that sleep problems modify the body’s inflammatory state and this modification is one of the main causes of cardiopulmonary, vascular-metabolic, and musculoskeletal diseases [23]. This modification involves an increase in the levels of circulating inflammatory cytokines, which would lead to excessive inflammation levels [67]. Lack of nocturnal sleep activates inflammatory signaling pathways, including nuclear factor-κB (NF-κB), activator protein-1 (AP-1), and transcriptional activator and signal transducer (STAT) family proteins. In addition, there is an increase in the levels of mRNAs that encode pro-inflammatory cytokines and an increase in monocyte production of interleukin-6 (IL-6) and tumor necrosis factor-α (TNF-α) stimulated by receptor 4 of the Toll type (TLR4) [68]. However, more persistent periods of sleep loss seem to be necessary for such inflammatory consequences to occur [68].

Despite the advance in knowledge in this area, the mechanisms that link sleep duration with the presence of multimorbidity are still poorly understood. It has been hypothesized that the presence of depression and low physical activity levels may contribute to this association [63, 64]. Short sleep duration is often combined with other behavioral risk factors such as smoking, excessive alcohol consumption, and physical inactivity, which can, in turn, increase the risk of chronic diseases [23]. Recent findings indicate that sleep influences hormones that control appetite, glucose homeostasis, and cortisol levels [32]. Therefore, it can be inferred that sleep has a great impact on the development and course of diseases of cardiopulmonary, vascular-metabolic, and musculoskeletal patterns.

Although the present study addresses sleep problems as an exposure and multimorbidity patterns as an outcome, there may be a bidirectional relationship between these variables, as reported in a previous study [30]. It is important to recognize the bidirectional relationship between sleep problems and multimorbidity in older adults and to implement measures to prevent and treat both problems. Strategies to promote healthy sleep, such as having a regular sleep routine, exercising, and avoiding excessive use of electronic devices before bed may be useful to improve sleep quality and reduce the risk of developing or worsening chronic health conditions in older adults.

The current study has some limitations that should be highlighted, such as the measurement of variables through self-report, which is a very common method used in epidemiological studies. However, the information is subject to memory and social desirability bias, which may result in underestimation or overestimation of association estimates. In addition, the typologies of sleep problems were investigated together (initial, intermediate, and final insomnia), but not in isolation, making a direct comparison with literature on such sleep problems impossible. Finally, the cross-sectional nature of the research imposes a limitation on the determination of the temporality of the associations found. Therefore, reverse causality is possible. On the other hand, the present study has strengths that include mainly the large sample size, substantially contributing to the generalization of the findings.

As implications of this study, our findings provide support for the recommendation of screening and early identification of sleep problems in older adults, aiming to prevent the occurrence of non-communicable chronic diseases, including the different multimorbidity patterns. It is necessary to understand the potential impact of sleep problems on older adults’ health in order to assist in the development and implementation of clinical and public health guidelines for the prevention and treatment of this important health problem. We strongly believe that better approaches to this condition in primary care should be considered so that this problem can be adequately absorbed by the health system.

## Conclusion

Sleep problems are independently associated with the investigated multimorbidity patterns. These results suggest that public health programs aimed at preventing sleep problems in older adults are essential to reduce possible adverse health outcomes, including multimorbidity patterns and their negative consequences on older adults’ health.

## Data Availability

The datasets used and/or analyzed during the current study are available from the corresponding author upon reasonable request.
